# Combining satellite geophysical data with continuous on-site measurements for monitoring the dynamic parameters of civil structures

**DOI:** 10.1038/s41598-022-06284-7

**Published:** 2022-02-10

**Authors:** Stefania Coccimiglio, Giorgia Coletta, Erica Lenticchia, Gaetano Miraglia, Rosario Ceravolo

**Affiliations:** 1 Department of Structural, Geotechnical and Building Engineering, Politecnico Di Torino, Corso Duca degli Abruzzi 24, 10129 Turin, Italy; 2 Responsible, Risk, Resilience Interdepartmental Centre (R3C), Politecnico Di Torino, Viale Mattioli, 39, 10125 Turin, Italy

**Keywords:** Civil engineering, Natural hazards

## Abstract

One key issue in the Structural Health Monitoring (SHM) of buildings is the influence of the soil on the dynamics of the system. The lack of accurate information on soil-structure interaction represents a source of significant uncertainty and generates difficulties in assessing the state of structural health. In this respect, satellite data could represent a valuable tool for soil knowledge. This paper presents the first study of satellite data coming from the environmental Copernicus program of the European Space Agency (ESA) for the alternative application in the field of SHM. In particular, Land Surface Temperature (LST) and Soil Water Index (SWI) data are elected to study surface temperature and moisture condition of the soil. Once examined and processed, these records have been statistically analyzed, crossed with on-site experimental quantities (natural frequencies and environmental variations), and given as input to a Finite Element (FE) model. The final goal is to understand the actual structural behavior, but also to monitor the evolution of the dynamic parameters for the purposes of structural and seismic monitoring. The largest oval masonry dome in the world was chosen as a prominent case study to demonstrate this novel approach to SHM.

## Introduction

In recent years, earthquakes and other catastrophic events have increasingly highlighted the fragility of the built environment. Infrastructures, buildings, and entire urban areas have proved particularly vulnerable to natural phenomena, whether caused by climate change (such as floods and landslides) or earthquakes, but also to human-made hazards. In this context, Structural Health Monitoring (SHM) systems can effectively contribute to the real-time assessment of a building, especially as they allow the detection of structural anomalies that may indicate damage.

Although the advantages of permanent monitoring systems are well known^1,2^, the high costs have not so far allowed widespread use on buildings. Currently, only some relevant and strategic structures are equipped with systems that jointly monitor multiple parameters, capturing the overall health of the system^3–5^. All structures, in fact, exhibit significant variations, or ‘wanderings’, of their dynamic characteristics as a consequence of real structural modifications or mere environmental fluctuations, which makes the problem of reliably inferring damage from such deviations^6–8^. Consequently, it is essential to investigate new strategies capable of guaranteeing, and possibly improving, continuous and systematic monitoring of buildings and infrastructures, especially to reduce their vulnerability to catastrophic events.

An innovative opportunity is represented by the use of satellite remote sensing data, which are becoming increasingly relevant in observing various phenomena. Satellite data are often used for environmental monitoring (i.e., melting glaciers, fires, drought, etc.), and the employment of these data for SHM is extremely recent; in particular, the satellite data of an interferometric nature are among the most widespread in this area, capable of detecting centimetric displacements even up to millimeters. Their first applications concerned the monitoring of aggregated buildings in urban areas^3,9–13^, the effects of land subsidence in built environments^3,14,15^, and only later the detection of anomalies on specific structures^16^, or infrastructures^4,11,16,17^.

It is worth pointing out that these applications are limited to the employment of only interferometric data obtained from radar instruments, e.g., Synthetic Aperture Radar (SAR). The literature in civil SHM does not report applications of satellite data of a different nature, such as the geophysical data coming from multispectral and hyperspectral sensors: in this research, the application of these data for SHM purpose is examined for the first time. In particular, the following aspects are addressed: (i) the data are selected, examined, and processed in view of their possible use in the context of SHM; (ii) a first study about the connection between structural, environmental, and geophysical satellite data has been performed; (iii) some preliminary Finite Element (FE) simulations that merge the information coming from in-situ experimental, environmental and satellite geophysical data have been performed in order to predict the complex dynamic behavior in operational conditions of a selected case study. The benchmark is the Sanctuary of Vicoforte, located in Piedmont, Italy, also known for its imposing masonry oval dome. The building is monitored by a permanent structural monitoring system, both static and dynamic^18^.

**Figure 1:**
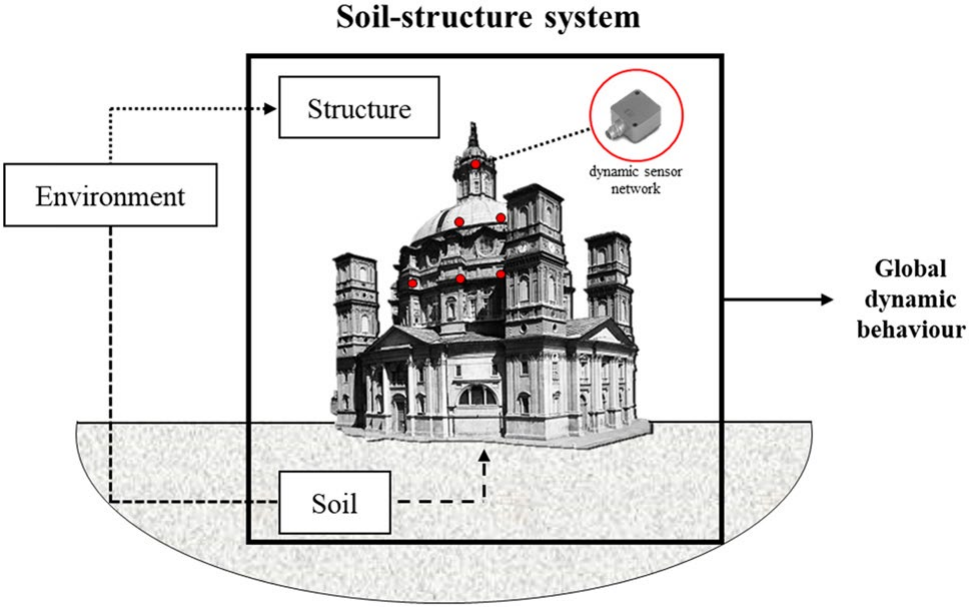
Scheme of soil-structure interaction and dependencies on environmental phenomena. (The figure was created by the authors with Microsoft Power Point^25^ software).

The geophysical information relating to the foundation soil of a structure could be of great interest for the SHM of buildings. In fact, although it is known that some environmental phenomena affect the dynamics of the system *directly*^4,19,20^, i.e., directly impacting the emerging structure, their *indirect* effect, represented by the modification of the geotechnical properties of the foundation soil in operational conditions, has never been studied in detail. It is equally relevant for SHM as an important variation of the soil parameters could lead to an evolution of the constraint conditions of the structure and, therefore, its dynamic behavior (Fig. 1). These fluctuations in dynamic diagnostic parameters, which can be caused directly or indirectly by phenomena such as rain, temperature variation, humidity, etc., are in most cases harmless. However, their recognition is important because, in the data analysis phase, they can be mistaken for structural damage (*false positive*) or, in the worst cases, hiding them (*false negative*)^21–24^.

The analysis of the indirect effects is made even more complicated by the limited availability of continuous monitoring data referring to the soil and the limited knowledge of the soil-structure interaction. In this study, the authors evaluated the use of Copernicus geophysical satellite data provided by the European Space Agency (ESA) and commonly used for environmental monitoring, as a source of continuous information about the foundation soil of a civil structure, to support in situ SHM. In addition to a systematic evaluation aimed at investigating whether or not satellite data contain useful information for our purpose, a technical-logistic appraisal of their usability in this sense will be addressed, i.e., evaluating whether their acquisition characteristics (spatial resolution, sampling frequency, percentages of missing or anomalous data) are suitable for the intended purpose, or can be made so through appropriate processing techniques.

In the following paragraphs, the involved data are briefly described. Further details are found in the literature^26–32^.

### Land surface temperature (LST)

The LST parameter is defined as the measure of the effective radiant temperature of the Earth surface or as the thermal radiance emitted from the land surface, as respectively reported in two studies^27,33^. It is obtained through a Split Window (SW) algorithm^34,35^ that takes into account the thermal radiation measured by the Sea and Land Surface Temperature Radiometer (SLSTR) of Sentinel-3 satellite (characterized by a near-polar and sun-synchronous orbit^36^) with an about 1 km spatial resolution, which provides multispectral measurements^34,37^. Considering this, the LST can also be defined as the effective radiometric temperature of the ground surface in the SLSTR field of view^35^. Additional parameters assumptions used for the data acquisition and processing can be found in the dedicated literature^35^.

### Soil water index (SWI)

The SWI parameter describes the soil moisture conditions in the first meters of depth, assuming values between 0 and 100%. It is obtained from instruments installed on the Sentinel-1 satellite (characterized by a near-polar and sun-synchronous orbit^38^) and Metop satellite (characterized Low Earth Orbit (LEO)). It can be defined as a percentage of saturation and is linked and driven by precipitation through the infiltration process^39^. SWI data have a daily sampling time and a spatial resolution of 1 km.

Eight SWI values and eight QFLAG values are available, depending on the characteristic time length *T*, which can assume a value of 2, 5, 10, 15, 20, 40, 60, and 100. *T* should relate SWI and soil depth, but it must be pointed out that the algorithm for the calculation of SWI does not take into account the stratigraphy and soil properties. In this paper, reference will be made only to *T* = 100, as it is the recommended value provided in the SWI validation report^40^ to be used when further information is not available for the specific case study.

Also in this case, the parameters used for the data acquisition and processing can be found in the dedicated in literature^41^.

### Environmental and in-situ SHM data

The environmental data, i.e., the average temperature records, hereinafter referred to as $\theta$_*air*_*,* for the case study of the Sanctuary of Vicoforte is provided by the database of ARPA Piemonte^30^. In particular, they are recorded from the station of Mondovì, CN, about 8 km away from the Sanctuary.

The dynamic monitoring data, in particular the natural frequencies of the structure, derive from the acquisitions of the permanent dynamic monitoring system installed on the Sanctuary^32^, whose data are analyzed in continuous time using operational modal analysis techniques^31,32^. Since deviations in the modal parameters are indicative of the condition state of a structure, their identification is a crucial phase of SHM, which is made more and more effective by the refinement of consolidated techniques^42^. In the analysis implemented in the present paper, the first two natural frequencies, *f*_1_ and *f*_2_, were considered. They correspond to the first and second translation modes of the system in the direction of the minor and major axis of the oval dome, respectively. It was decided not to involve the other natural frequencies since, in the specific case of the Sanctuary, the selected frequencies have a much higher identification percentage^31^ than the higher ones allowing stable observations. Regarding the consistency with the satellite data acquisitions and their sampling, the identification of the accelerometric signals acquired at about 12:00 a.m. was used. In the following sections, the intersection of the different types of data was made with reference to the period 02/18 to 01/19, for which all the series are complete.

**Figure 2:**
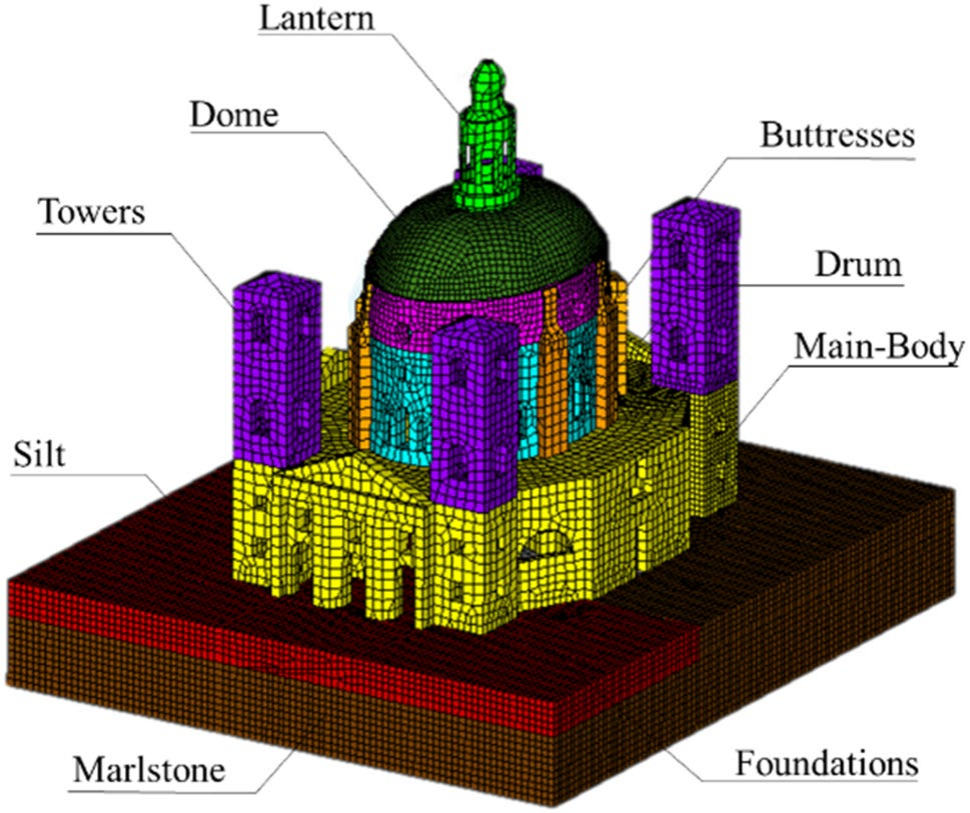
FE model of the Sanctuary of Vicoforte and macro-elements. (The figure was created by the authors with DIANA FEA^44^ and Microsoft Power Point^25^ software).

The parameters derived from the identification (performed on signals acquired at the temperature of 10 °C) were also used for the calibration of the FEM of the structure. Thanks to the calibration, the model, shown in Fig. 2, reproduces the actual dynamic behavior of the Sanctuary and can be used to evaluate the response of the system to changes in the conditions imposed by the user^43^.

## Results

**Figure 3:**
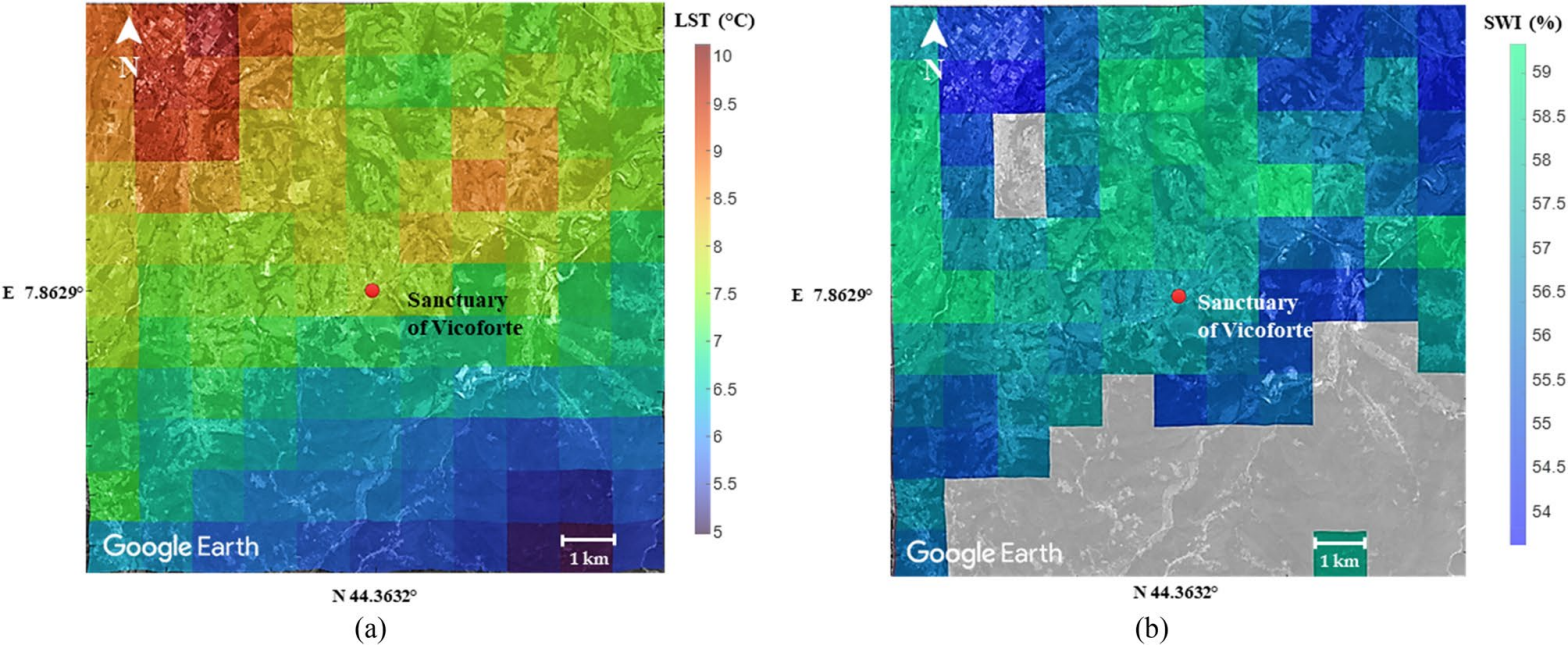
Two-year (2018–19) average data interpolated on 121 points in the 11 km × 11 km grid around the Sanctuary superimposed on Google Earth view: LST_original_ (**a**) and SWI (**b**) data. (The figures are created with the use of Google Earth^47^, MATLAB 2020b^48^ and Microsoft Power Point^25^).

After having independently analyzed the satellite data and processed them for the purpose of this research, as described in the “Methods” section, they were crossed with the time series of the first two structural frequencies of the Sanctuary in order to investigate possible relationships. It is worth highlighting that these measurements contain information about different phenomena: SWI is known to be related to precipitation, which, in addition to modifying the properties of the soil, could lead to an increase in mass and a consequent alteration of the structural frequencies by wetting the material of the overlying structure; similarly, the environmental changes in temperature affect the structure and therefore its frequencies both directly and indirectly, e.g., by modifying the mechanical properties of the ground. Separating all these effects is not trivial, even considering the fact that some relations are non-linear. For greater clarity, the influence of the mechanical parameters of the ground on the dynamics of the system is not questioned here, as it is already a fairly consolidated concept^45,46^, but rather, the possibility of monitoring soil properties within the series of geophysical satellite data considered and therefore of their usability for SHM is evaluated. For this precise purpose, the satellite data were collected and, to correct the sampling not always coherent in space, interpolated at predetermined points on a grid of 11 × 11 km around the point of interest (Fig. 3).

The maps of LST and SWI in the grid around the Sanctuary (two-year average data) show that, in general, the LST is higher in the built-up areas (North-West of the net, corresponding to the town of Mondovì) and lower in the areas where the vegetation is denser (South of the net). This last area appears to be missing SWI data, probably due to the high slope of the surface. The Sanctuary is located in the intermediate area with an average value between 7 and 8 °C for LST and 57% for SWI.

**Figure 4:**
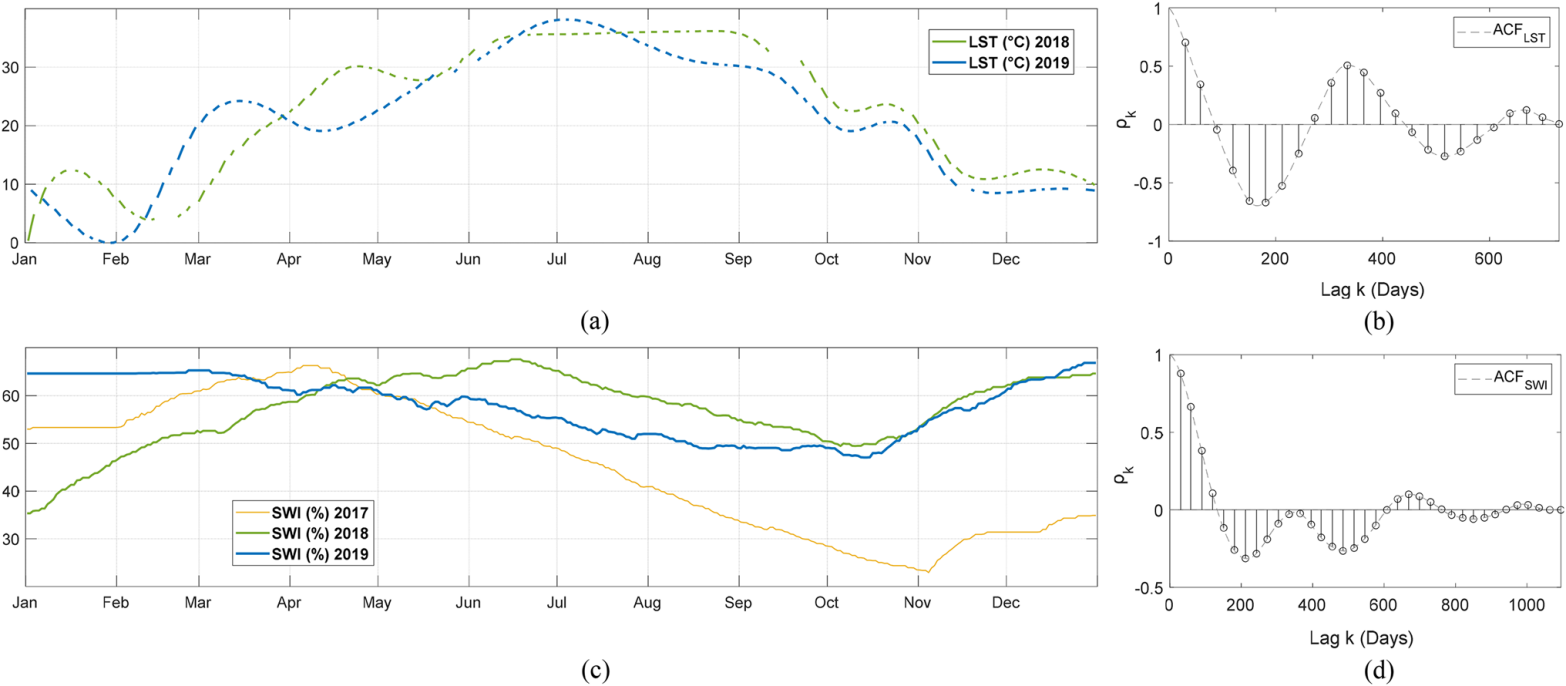
Satellite data autocorrelation analysis: overlapping of annual LST series (**a**), LST ACF (**b**), overlapping of annual SWI series (**c**) and SWI ACF (**d**).

Data have even been involved in an autocorrelation study whose results are shown in Fig. 4.

In the graphs on the right, the total signals of LST and SWI (which includes 2 and 3 years of measurements, respectively) were divided into years and superimposed: from these, it can be appreciated that LST seems to trace almost the same trend over the years analyzed (Fig. 4a), an aspect confirmed by the shape of the autocorrelation function (ACF), which shows a peak of the autocorrelation coefficient *ρ*_k_ when the total signal is shifted by about 365 days (Fig. 4b); LST also shows a clear seasonal trend, which increases in summer and decreases with the arrival of winter. The same thing does not seem to happen for the SWI parameter. In the first phase, for consistency with LST, the SWI data had been collected and analyzed since January 2018. However, the important disparity between the values of the first months of 2018 and 2019 led to searching for the cause in the previous records, and therefore, the entire 2017 series was also involved in the analysis. In each year, there were high SWI values in the spring and dips between the months of October and November, with the subsequent ascent (Fig. 4c). The peculiarity of 2017, which also affects the values at the beginning of 2018, is in the value of that autumn abatement, which is about half when compared to the following years. This was probably due to the period of low rainfall between the summer and autumn of 2017^49^, interrupted in the first week of November when the SWI ascent begins. This close dependence on rainfall, which is partly linked to seasonality and partly to less recursive environmental phenomena, makes this index less cyclical than the LST, and therefore the total signal is less autocorrelated. This is evident in the ACF, which shows no peaks at around a 1-year lag (Fig. 4d).

**Figure 5:**
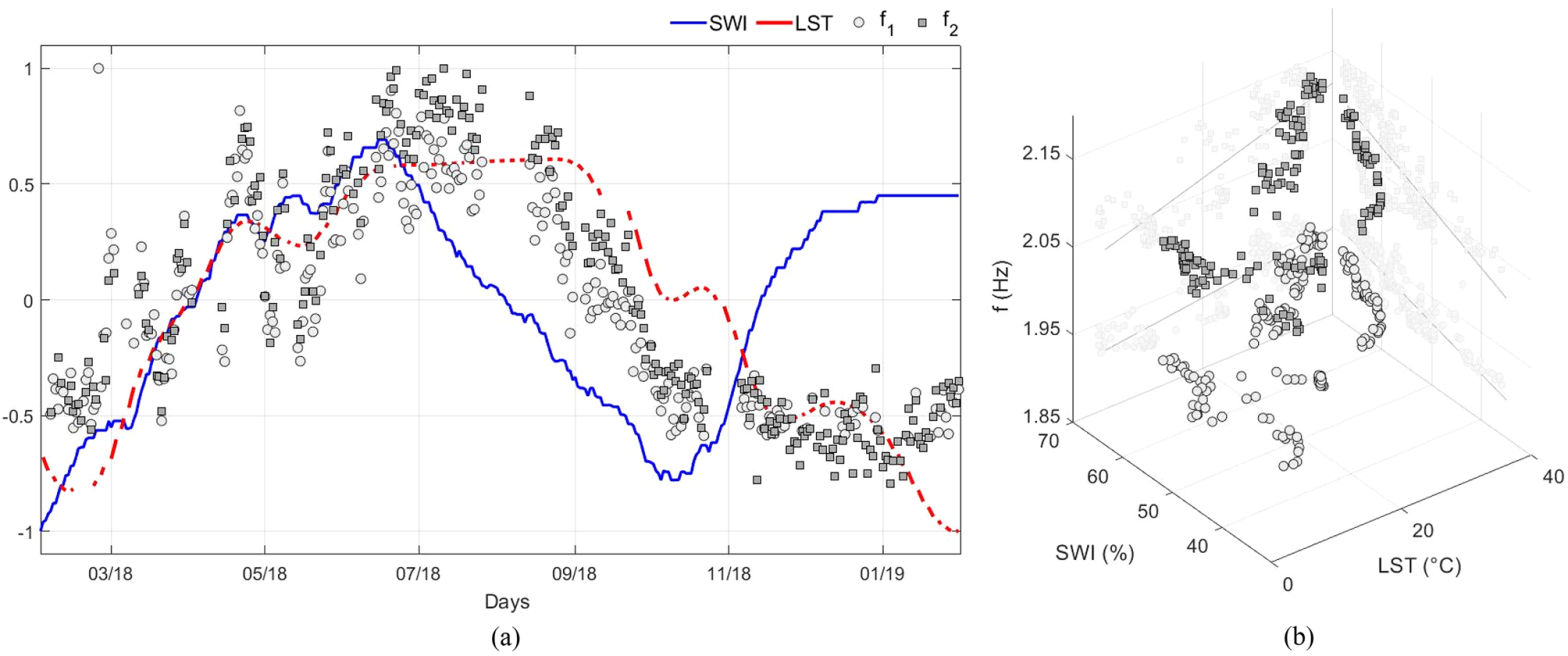
Overlapping normalized time series: LST, SWI,* f*_1_ and* f*_2_ (LST is represented by a solid line: interruptions are due to invalid measurements) (**a**); 3D scatterplot between LST, SWI and frequencies (**b**).

In Fig. 5, the crossing of the 2018 time series of satellite and frequency data are shown. As anticipated, the first two natural frequencies of the Sanctuary were considered, *f*_1_ and *f*_2_.

As satellite data have been cleansed to remove fast fluctuations (see “Methods” section), the figures have the sole purpose of analyzing a long-term trend.

The graphs in Fig. 5a show that the LST increases in the warmer months, as do frequencies^32^, thus seem to be somehow related. This outcome agrees with what was expected, given the strong relationships found between LST–$\theta$_*air*_ (see their correlation coefficient in Table 1) and $\theta$_*air*_–*f*^32^. It is also appreciable a difference between the frequencies and LST at the beginning and at the end of the series when the temperatures are more rigid: it is probably due to the bilinear trend of the frequencies^32^ with the temperature, which inverts the slope for values lower than 0 °C. As for SWI, it tends to grow for the first half of the year examined, this being in accordance with the dynamic parameters. However, starting from mid-June 2018, it anticipates the descent with respect to the other observed parameters. Moreover, from mid-October onwards, the trends seem to diverge, contrary to what happened in the first half of the year.

**Table 1:** Linear correlation coefficients of LST, SWI, f_1,_ f_2_ and $\uptheta$_air_.

	*f*_1_	*f*_2_	$\theta$_air_
LST	0.7807	0.8707	0.9433
SWI	0.4384	0.1946	0.1101

Globally, while in the first half of the year all the series seem to grow quite consistently, in the autumn the frequency trends deviate from both series of satellite data. In particular, it is observed that the seasonal decrease in frequencies anticipates that of LST (and of the ambient temperature) and instead lags behind the beginning of that of SWI. This could be due to a dependence not exclusively on one of the effects represented by the satellite parameters but on both: soil temperature and soil moisture. To better visualize the relationship between the collected measurements, they were represented in a 3D scatterplot (Fig. 5b), and for each pair, the linear correlation coefficient is calculated and reported in Table 1.

The correlation reaches a value of 78% and 87% for *f*_*1*_ and *f*_*2*_, respectively, both greater than the corresponding coefficients calculated between frequencies and $\theta$_*air*_ (72% and 75%), as reported in^32^. As regards SWI, equally significant values are not reached. Theoretically, what one would expect from the relationship between SWI and the frequencies of the structure would be an inversely proportional relationship. In fact, as also demonstrated in^50–52^ the variations in soil stiffness show a non-linear relationship with the degree of saturation, which in general implies a non-linear decrease of shear stiffness when the degree of soil saturation increases. However, this behavior does not seem to happen in the dataset analyzed here, where positive correlation coefficients between frequencies and SWI are obtained: this aspect deserves in-depth analysis aimed at modeling the relationship between the degree of saturation and the stiffness of the soil to be validated on experimental frequency data suitably purified of the effect of temperature.

**Figure 6:**
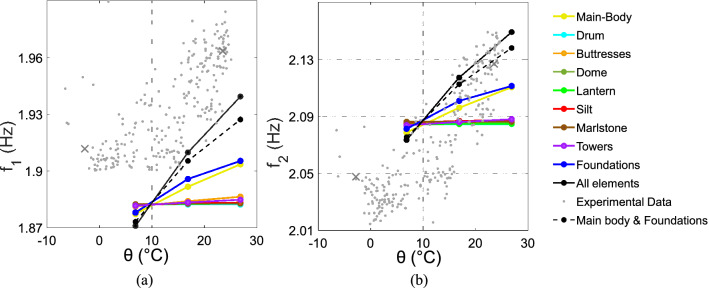
Frequency-temperature relation for each macro-elements, for main body and foundations together and for all structural elements:* f*_1_ and $\uptheta$ (**a**),* f*_2_ and $\uptheta$ (**b**).

Given the greater complexity of the aspects related to the degree of soil saturation and its stiffness, which will deserve a separate study, in the last part of the research, we have limited ourselves to the effect of temperature. By simulating the thermal variation in the FE model of the Sanctuary, both on the emerging structure and on the soil, it has been possible to investigate the influence of the temperature on the dynamic response of the system (Fig. 6).

Before commenting on the results, it should be clarified that the lack of overlapping of the numerical and experimental data is only linked to the residual calibration difference, which does not represent a problem since here the focus is on the frequency-temperature dependence, rather than on their values absolute. The graphs show that most of the lines tend to be horizontal; this indicates that the single change in temperature of the associated elements (drum, buttresses, dome, lantern, silt, marlstone, towers) slightly affects the frequencies of the structure. By applying the temperature variation to the whole FE model simultaneously, the relation assumes a very sharp trend and tends to follow the slope of the experimental data. The elements that individually correspond to the most marked changes in frequency are the foundations and the main-body: a further analysis was performed by applying the temperature variation to both together (dashed black line in Fig. 6) in order to compare it with the analysis which implies the variation on all elements (solid black line in Fig. 6). The result shows that the frequency trends are very close but do not overlap. This means that the two elements at the base of the Sanctuary are the main, but not the only ones, responsible for the dependence of the dynamic response on temperature.

## Discussion

The research presented in this article introduces satellite geophysical data from an SHM perspective for the first time. This discussion will cover the following aspects: analysis of satellite data for SHM, selection of strategies to make them suitable for monitoring of buildings, statistics, pros and cons, possible developments.

Both LST and SWI data present high spatial variations that can be easily perceived from observing a relatively small analyzed surface (Fig. 3). This can be considered an advantage on the one hand because it means being able to work with accurate data, but, also considering the inconstant spatial sampling, great care must be taken in choosing the point of interest if one wants to use this data for structural monitoring instead of environmental monitoring, for which they were designed. A possible general solution to mitigate any local anomaly, and make the time series more reliable and robust, is the spatial interpolation that has been implemented between the coordinates closest to the observed point. The interpolation in this specific case was deemed reliable also because the annual average values of the parameters in the points surrounding the Sanctuary are very close (Fig. 3), but this is not always guaranteed. As regards the reference system, the standard geodetic system for this type of geophysical satellite data has been assumed, i.e., the WGS84.

For the coordinates of the Sanctuary, the LST presents a trend over time characterized by important fluctuations towards very low temperature values. Since for this research the LST parameter is employed as a measure of the soil surface temperature, and being evident that some temperature records (up to − 50 °C) are unrealistic for that latitude and altitude, the records need to be appropriately cleansed. Any data conditioning implies a certain degree of uncertainty as it makes the trend very smooth, cleansing the series also of fast variations that could be real. Although Hilbert transforms and filtering operations may represent a valid solution to eliminate unrealistic values, their parameters should be carefully calibrated on a case-by-case basis^53^.

The SWI parameter is strongly conditioned by the selection of the reference time length, *T*. Eight SWI values and eight QFLAG values are available, depending on the characteristic *T*, which can assume a value of 2, 5, 10, 15, 20, 40, 60, and 100^29^. In particular, it was noted that for very low *T*, SWI is very influenced by the rainfall trend. Also, in this case, this choice involves a considerable degree of uncertainty; although reference papers^40^ suggest *T* = 100 in cases where more precise information is not available, this may be questionable in the case of applying such data to structural monitoring. Even in the latter case, the most desirable solution would be to evaluate the optimal *T* case by case, perhaps based on the depth of the foundations of the monitored structure.

The cross between the frequency data with the satellite data highlighted the following aspects: LST seems to follow the same seasonal trend as the on-site monitoring data. Regarding the correlations found, it is worth clarifying two aspects: (i) the correlation coefficient, measuring the strength of a linear relationship, fails to capture the presence of (strongly) non-linear relationships: it must be captured by the visual examination of the scatterplot. However, for slightly non-linear relations, the coefficient can still be used as it can capture the order of magnitude of the correlation; (ii) correlation does not imply causation. This means that even if two of the parameters considered showed a high correlation, they could not be linked by a cause and effect relationship, but they could be both caused by the same cause without influencing each other (debate on spurious and non-spurious associations). For instance, with reference to the phenomenon of temperature variation, having verified the correlation between $\theta$_*air*_ −*f* and $\theta$_*air*_−LST, it would be natural to expect that *f* and LST are also correlated since they are two effects triggered by the same phenomenon, but this does not prove that changes in soil properties are themselves a cause of frequency wandering.

Indeed, despite the high correlation found between LST and frequencies, the FE model simulations highlight a weak dependence between the dynamics of the system and the temperature variation applied only to the ground, lower than that shown by applying the thermal change to the material of the structure. Surely one reason lies in the fact that the ground has a great thermal inertia and especially the deeper layers are subject to very low thermal variations during the year compared to the emerging structure, directly exposed to climatic phenomena. Moreover, we must specify that just the simpler effect of the temperature on the ground has been modeled. More complex effects, such as evaporation triggered by temperature and the consequent variation in saturation degree and mechanical properties of the soil, have not been considered here and would require further studies.

In addition, it is worth underlying that natural frequencies are global parameters of a system, which synthesize all the local characteristics of the structure components, such as material heterogeneity that in the present case may be possibly induced by non-uniformity in the temperature of materials of the Sanctuary: the consideration of this aspect could refine the results. The correlation between SWI and natural frequencies is not as close, but this does not exclude at all their connection. Clearly, the series of experimental frequencies that have been involved are the result of all the phenomena that influence the stiffness, among which the temperature (relating to the soil or the environment) stands out, as has been highlighted. This very important effect could have obscured the influence that soil saturation could exert on the ground so as to make it invisible in the scatterplot: in principle, this criticality could be resolved by investigating the evolution of the dynamic behavior of the Sanctuary as a function of the degree of saturation of the soil, keeping the temperature constant. While not technically feasible on a structure the size of the Sanctuary, it could also be simulated on the FE model in future research.

Furthermore, the choice of the time length, in our case *T* = 100, influences the search for the *f*-SWI relationship and it should be well thought out before drawing any conclusions on the independence of the parameters.

Despite the long list of challenges, satellite data have the potential to become a useful source to assess the health of a structure, especially for helping define structural early warning methodologies. A key aspect of this research is the application of satellite data for structural monitoring. The idea of this research lies in using satellite data to monitor the ground properties, which is the basis of a structure equipped with an in-situ monitoring system. The hypothesis that guides this research is that these data contain information related to the stiffness of the soil-structure system, therefore partly responsible for the variation of the dynamic parameters identified. If this were true, the simultaneous reading of the various time histories and the application of techniques developed within the SHM could be of great support to damage detection, as many of the variations of the diagnostic parameters (in this specific case, modal frequencies) could be justified with the variations of the above parameters. This would allow reconstructing in a very accurate way the dynamic behavior of the system in a "healthy" condition and, therefore, to immediately notice the appearance of an anomaly (damage).

Despite the limitations and uncertainties of this first application of satellite data illustrated in the previous paragraph, their use could bring some advantages in SHM: they represent a successful solution for continuous and systematic monitoring at a reduced cost as they can be collected quickly and easily through platforms at no charge, with availability within 1–3 days of acquisition for the entire Earth's surface^54^. Then, given their global coverage, it could be possible to obtain data for any building avoiding the high costs of soil periodicals in situ tests. Thus, after the initial effort in understanding and processing satellite data, the process could be automated and extended to many types of buildings. Moreover, they allow obtaining data without directly intervening on the monitored asset, respecting the requirement of non-invasiveness, essential, for example, in the case of monumental building. Satellite data represent a capable tool when data from standardized tests are not available, or these tests cannot be performed because of problems linked to the site configuration, even though lack of direct control on instruments could be disadvantageous in case of breakage or anomaly.

Future studies will have to focus on clarifying all aspects of this new application of satellite data in SHM and will have the aim to better model the link between the physical parameters at stake, even on several case studies. This would make it possible to validate these data and affirm their potential not only for monitoring of a particular building but generalizing the process for other structural typologies (e.g., infrastructures such as bridges or entire urban areas).

## Methods

### Data processing

The platform CREODIAS^55^ and VITO Earth Observation^56^ of Copernicus Global Land Service (CGLS) have been exploited to collect satellite data. They have been processed to make them more consistent for the purposes of the study. The measuring points are spaced about 1 km apart, and, in the case of LST, they do not remain constant in the various acquisitions. While this kind of sampling is quite exhaustive for the environmental monitoring, it may not be accurate enough for SHM where the area of interest is relatively small. To overcome these issues, the geo-located data were used to derive the values on a fixed squared network with a constant step of 1 km through a process of triangulation-based linear interpolation through a program specially written in a MATLAB 2020b^48^ environment (www.mathworks.com). This allows to get more robust and less sensitive data to possible anomalous measurements and to work with data related to consistent coordinates. This procedure was followed for all observations in the dataset for both satellite data, obtaining a time series for each point of the network. A much larger area than the footprint of the structure was processed in order to evaluate any differences compared to neighboring places (Fig. 3). For clarity, the time series are not directly available on the mentioned platforms: they have been reconstructed by rearranging the data of the various acquisitions to evaluate their temporal evolution, this being an aspect of fundamental importance for SHM.

**Figure 7:**
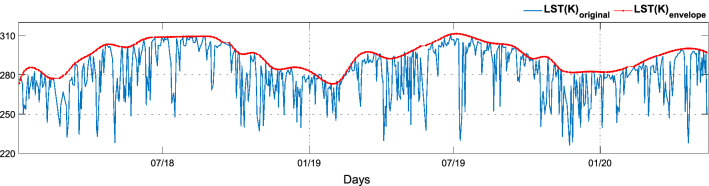
LST: original and enveloped signal.

After analyzing the area around the monitored structure, attention can focus more on the internal coordinates of the observed object. LST trend presents peaks with very low values corresponding to temperatures reaching up to − 50 °C. Although these values may find significance for other fields, they are deemed less significant for SHM because they refer to low values of the measurements used to derive LST rather than sudden drops in soil temperature. Moreover, due to the high thermal inertia, isolated thermal changes do not influence the dynamics of the structure appreciably. For these reasons, the data have been processed in order to draw the information contained therein useful for supporting on-site structural monitoring. The upper envelope of the LST original signal has been computed in order to neglect the negative peaks and, at the same time, keep the seasonal trend of the data. The signal envelope is obtained using a spline interpolation over local maxima divided by at least *n* samples, in this case, *n* = 10 (Fig. 7)^57,58^.

In subsequent analyses, only LST_envelope_ is considered, which for the sake of brevity will be referred to simply as LST. This proves necessary since satellite data are characterized by disturbances and, in this specific case, by sudden drops of temperature to values that are difficult or almost impossible to reach in the study area, thus being classified as artifacts or noise.

### Autocorrelation analysis

An autocorrelation analysis has been carried out^59^ in order to examine the trend over time of the two parameters. This analysis allows observing how similar the variables are to themselves after a certain period of time by comparing the signal at a given observation with another delayed value of itself and evaluating how much it correlates as time goes on. Considering different $k$ lag values, the autocorrelation coefficient ${\rho }_{k}$ for the general time series ${y}_{i}$ was calculated by the relationship:

1 $$\rho_{k} = \frac{{\frac{1}{N}\mathop \sum \nolimits_{i = 1}^{N - k} \left( {y_{i} - \overline{y}} \right)\left( {y_{i + k} - \overline{y}} \right)}}{{\sigma_{y}^{2} }}$$

where $N$ is the length of the series while $\overline{y }$ and ${\sigma }_{y}^{2}$ are its mean value and its variance. In the present case, given the available sampling, attention was paid to seasonal and annual changes. Results are shown in Fig. 4.

### Correlation analysis

In Fig. 5, remote sensing data were crossed with the on-site dynamic parameters: the series are overlapped by removing their mean and scaling to their maximum absolute value in order to focus the analysis on their fluctuations. The linear correlation coefficient, also with respect to $\theta$_air_, was also calculated as follows and reported in Table 1:

2 $$r\left( {x,y} \right) = \frac{{cov\left( {x,y} \right)}}{{\sigma_{x} \cdot \sigma_{y} }}$$

In which $cov(x,y)$ is the covariance, ${\sigma }_{x}$ and ${\sigma }_{y}$ are the standard deviation of $x$ and $y$ respectively. It measures how strong a (linear) relationship is between two variables, varying between values of |1| and 0. The higher the modulus of *r*, the stronger the relationship between the variables, while the sign indicates the nature of the bond (positive or negative correlation).

### FE simulations

Following the processing and analysis of the experimental data, the environmental and geophysical thermal conditions measured were introduced and applied on the FE model of the Sanctuary. As anticipated, the simulations are limited to the temperature data, as SWI will require a separate and more specific study.

The simulations aim to improve the interpretation of the dynamics of the system as a function of the thermal variations. To introduce these variations in the FE code, two approximate models were considered: one aimed to evaluate the distribution of temperature along the depth of the soil and the other to relate mechanical parameters to temperature. Soil and foundations temperature were estimated along the depth starting from the LST data. An approximate solution of the spatial damped diffusion Equation^60^, was derived:

3 $$\theta \left( {z,t} \right) = \overline{LST} + \Delta LST\left( t \right)\cdot e^{{ - \frac{\beta }{2\alpha }z}} \left[ {\cos \left( {z\sqrt {\frac{\lambda }{\alpha }\left( {1 - \frac{{\beta^{2} }}{4\lambda \alpha }} \right)} } \right) + \frac{\beta }{{2\alpha \sqrt {\frac{\lambda }{\alpha }\left( {1 - \frac{{\beta^{2} }}{4\lambda \alpha }} \right)} }}\sin \left( {z\sqrt {\frac{\lambda }{\alpha }\left( {1 - \frac{{\beta^{2} }}{4\lambda \alpha }} \right)} } \right)} \right]$$

where $\alpha >0$ and $\beta >0$ are material constants, and $z$ stands for depth ($z>0$ downward). In the solution of the diffusion equation, it was assumed $\frac{\partial \Delta \theta }{\partial z}\left(0,t\right)=0$, where $\Delta \theta$ is the absolute temperature amplitude (in Kelvin) around the mean value, while the amplitude of LST was imposed as a boundary condition at surface (*z* = 0) for any time. In (3) $\overline{LST }$ is the mean value of LST over the observed time,$\Delta LST\left(t\right)=LST\left(t\right)-\overline{LST }$, $\alpha$ is the thermal diffusivity, $\beta =2\sqrt{\pi \alpha /{D}_{0}}$^61^ with ${D}_{0}$ fundamental period of $\Delta LST$, i.e. the average time that elapses between two main peaks in $\Delta LST\left(t\right)$. Theoretically $\lambda =-\frac{\partial \Delta LST}{\partial t}\frac{1}{\Delta LST}$ and it has been set equal to the median value of theoretical instantaneous estimate after rejecting the negative results. It is possible to estimate ${D}_{0}$ from experimental data as the inverse of the main frequency picked in the Fourier spectrum of $\Delta LST\left(t\right)$.

The temperatures of the various layers of soil have been estimated as the average values between the thickness of the layers used to discretize the soil in the FE model:

4 $$\theta_{{z_{i} z_{i + 1} }} \left( t \right) = \frac{{\mathop \smallint \nolimits_{{z_{i} }}^{{z_{i} + 1}} \theta \left( {z,t} \right)dz}}{{z_{i + 1} - z_{i} }}$$

where ${z}_{i+1}$ and ${z}_{i}$ are the boundaries coordinates of the soil layer *i* (${z}_{i+1}>{z}_{i}$)*.*

**Figure 8:**
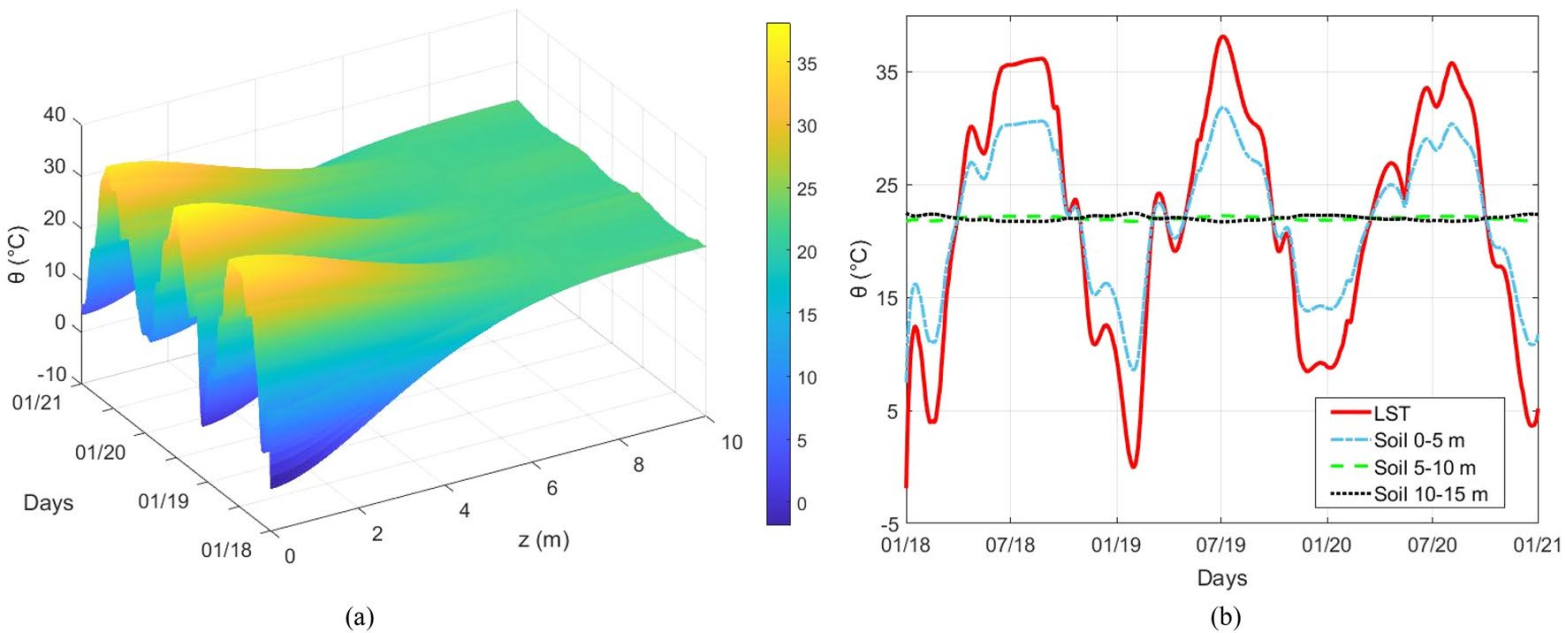
Time-depth distribution of soil temperature (**a**); mean temperature at different depth (**b**).

For the case study of the Sanctuary of Vicoforte, the value of thermal diffusivity in Eq. (3), has been obtained exploiting the temperature at *z* = 3.6 m recorded during the inspection of December 17, 2020: the experimental read of 15 °C for those specific values of *z* and time is used to find $\alpha$, which minimizes the error in the prediction with (3). The minimization is carried out on a range of thermal diffusivity values between 0.1e−7 m^2^/s and 100e −7 m^2^/s with step 0.1e−7 m^2^/s, in accordance with the typical values of the literature for marlstone and silt^61,62^ and using the normalized error as cost function. The values of $\alpha$ obtained from the minimization process are reported in Table 2 together with the other estimated values. Figure 8 shows the results of the temperature-depth model. For the minimization operation, the LST data up to December 2020 were employed, given the date of the inspection.

**Table 2:** Temperature-depth model parameters.

*D*_*0*_ [s]	λ[1/s]	α[m^2^/s]
2.7034e + 07 (about 312 days)	2.2451e−07	9.8000e−07

At this point, having the temperature of the soil and the macro-elements, a simplified model is used to apply the temperature variations to the mechanical parameters of the FE model.

The simplified model for the Young’s modulus $E(\theta )$ and temperature $\theta$ is reported below:

5 $$E\left( \theta \right) = \left( {E_{0} - r\theta } \right)\left[ {1 + \left( {\alpha_{l} \left( \theta \right) - \alpha_{s} \left( \theta \right)} \right)\theta } \right]$$

where ${E}_{0}$ is a fictitious zero Kelvin Young’s modulus, it is defined as fictitious because the law is linearized at small relative temperatures; $r$ is the tangent of Young’s law of modulus which ideally describes the effects of thermal agitation at low relative temperatures (i.e. around 273.15 K). ${\alpha }_{l}(\theta )$ and ${\alpha }_{s}(\theta )$ represent the thermal expansion coefficient as a function of absolute temperature $\theta$, where the subscripts *l* and *s* stand respectively for liquid and solid. Usually, we can assume ${\alpha }_{l}(\theta )-{\alpha }_{s}(\theta )\sim {\alpha }_{l}(\theta )$ since ${\alpha }_{l}\left(\theta \right)\gg {\alpha }_{s}(\theta )$. In this study, as a first approximation, the contribution of thermal agitation in the variation of the Young’s modulus with environmental temperature values ​​is considered negligible and, therefore, $r$ is assumed to be zero. Finally, since the liquid phase in masonry (as in other materials) consists mainly of water, the law becomes:

6 $$E\left( \theta \right) \approx E_{0} \left[ {1 + \alpha_{H2O} \left( \theta \right)\theta } \right]$$

with *α*_*H2O*_($\theta$) thermal expansion coefficient of water. For the latter, in the case of $\theta$>273.15 K the values ​​reported in https://webbook.nist.gov/chemistry/ have been applied to exactly fit a polynomial of a high order, while in the case of $\theta$<273.15 K an average fit with the same polynomial was used since for temperatures below of 0 °C to − 50 °C the water in the pores can coexist in liquid and solid phase^63^, therefore, on a macroscopic scale, the coefficient of thermal expansion will adapt to an intermediate state. The coefficient is characterized by a high variation around 0 °C due to the phase transition between liquid water and ice. However, it should be noted that this model has not yet been experimentally validated.

Knowing the temperature of both the soil and the masonry, through Eqs. (4) and (6), it was possible to obtain $E(\theta )$ of each element. The maximum, minimum, and average temperatures recorded in 2018 were considered for consistency with the experimental frequency data. Results are shown in Fig. 6. In these simulations, only the temperature effect is considered, neglecting all other phenomena that can influence the dynamics of the structure.
